# Leptospirosis, a Re-emerging Threat

**DOI:** 10.7759/cureus.14295

**Published:** 2021-04-05

**Authors:** Margarida Brito Monteiro, Inês Egídio de Sousa, Maria Piteira, Sílvia Coelho, Paulo Freitas

**Affiliations:** 1 Internal Medicine, Hospital Professor Doutor Fernando Fonseca, Amadora, PRT; 2 Internal Medicine, Hospital São Francisco Xavier, Lisbon, PRT; 3 Internal Medicine, Hospital do Espírito Santo de Évora, Évora, PRT; 4 Intensive Care Unit, Hospital Professor Doutor Fernando Fonseca, Amadora, PRT

**Keywords:** zoonosis, leptospirosis, liver dysfunction, kidney dysfunction

## Abstract

Leptospirosis is a zoonosis transmitted by an animal vector and caused by the spirochete bacteria Leptospira. Human infection is rare and acquired by exposure to environmental sources (animal urine, contaminated water, soil, or infected animal tissue). It can have an extremely broad presentation, ranging from asymptomatic to serious illness.

We report the case of a 59-year-old man admitted to the hospital with myalgia, fever, and abdominal discomfort. Routine laboratory tests revealed raised inflammatory markers, thrombocytopenia, kidney dysfunction, and hepatic cytolysis and cholestasis. The hypothesis of zoonosis was raised based on symptoms and analytical changes despite the weak epidemiological history to support it. Although leptospira serology tests were negative on admission, a polymerase chain reaction test was requested due to a high degree of suspicion which came back positive. The patient completed eight days of doxycycline with favorable clinical and analytical progression.

This case highlights the changing epidemiology of leptospirosis and the importance of having a high degree of suspicion even outside endemic zones. It also highlights the importance of a wise choice of diagnostic tests according to the disease stage.

## Introduction

Leptospirosis is a re-emerging zoonosis with a high worldwide distribution. It is one of the most widespread and prevalent zoonotic diseases. Climate change, human behavior, animal husbandry with the urbanization of some rural areas, and the increased contact with wildlife species lead to the growth of overall cases and it can no longer be considered a rural disease [1]. Human infection is accidental after direct or indirect exposure to contaminated environments (soil, water, or surfaces) by the urine of leptospirosis-infected animals. The spectrum of the disease is extremely wide and it can make the diagnosis difficult. A combination of nonspecific laboratory findings can only suggest leptospirosis. Immunoglobulin M (IgM) enzyme-linked immunosorbent assay (ELISA) is normally used as the standard serologic procedure but the polymerase chain reaction (PCR) test has the ability to assure a definite diagnosis during the acute phase. As prompt treatment has an impact on associated mortality, an early diagnosis is vital to reduce the progression and severity of the disease. An early antimicrobial therapy together with prompt supportive therapy can drastically reduce the mortality rate.

## Case presentation

The case refers to a 59-year-old man, a construction worker, with a past medical history of hypertension and moderate alcohol intake. He lived in an urban area and denied recent travel or animal contact. However, he confessed to having some contact with a small rural area close to home.

He was seen in the emergency room with a five-day history of fever and myalgia (predominantly in the lower limbs and lumbar region) worsened then by vomiting and abdominal discomfort. On admission, vital signs and physical examination were unremarkable.

Blood tests revealed anemia (11.9 g/dl), raised inflammatory markers (leukocytosis 10300 /L and C-reactive protein 37.7 mg/dL), thrombocytopenia (56000 /L), acute kidney injury (creatinine 2.39 mg/dl to a baseline of 1.25 mg/dl), raised creatinine kinase (CK) suggesting rhabdomyolysis (CK 8288 U/L), hepatic cytolysis and cholestasis (TGO 291 UI/L, TGP 99 UI/L, GGT 101 UI/L, total hyperbilirubinemia 1.46mg/dl) and raised lactate (3.24 mmol/L). Urinalysis showed proteinuria (150 mg per day) and haematuria. Renal ultrasound was normal.

Assuming sepsis with multiorgan dysfunction, blood cultures were collected as well as bacterial and viral serologies were performed. Fluid therapy was initiated with an improvement of the lactate levels (3.24 > 1.9 mmol/L) and rhabdomyolysis. Despite that, the patient developed a rapid deterioration with worsening kidney and liver function as well as haematologic impairments. He was admitted to the intensive care unit (ICU) and started on invasive mechanical ventilation, vasopressor support, and continuous renal replacement therapy.

The hypothesis of zoonosis was raised based on symptoms (flu-like) and the combination of the laboratory findings (especially thrombocytopenia and liver and kidney dysfunction) despite the weak epidemiological history. Empirical antibiotics were started with doxycycline and meropenem. Blood cultures, as well as bacterial and viral serologies, were negative including leptospire-specific IgM by ELISA. Due to a high degree of suspicion, a real-time PCR test for leptospirosis was performed which came back positive, thus confirming the diagnosis. He completed eight days of doxycycline with favorable clinical and analytical progression. Unfortunately, serologies were not repeated during hospitalization. He was discharged on day 14 with no sequels.

## Discussion

Leptospirosis is caused by spirochete bacteria of the genus Leptospira with several pathogenic species and serovars. Indirect exposure accounts for most sporadic cases [2]. Although leptospirosis is more common in developing countries, there is an increasing prevalence in the last few decades in Portugal (particularly in the central mainland and Azores) with the occurrence of fatal cases [2].

It ranges from a subclinical infection (approximately 90%) [3] to a severe syndrome with multiorgan dysfunction (the classic Weil’s disease) that can be fatal, mainly if not treated. The onset of clinical illness occurs abruptly after an incubation period of 2-30 days, although most symptoms occur 5-14 days after exposure [3]. The disease can be divided into two stages of symptoms. An initial phase (acute phase/ septicemic) starts quickly and lasts 5-7 days characterized by unspecific symptoms that can mimic mild flu (fever, myalgias, headache) after which the patient may become relatively asymptomatic. The second phase occurs when antibodies appear in the serum and leptospires are excreted in the urine (immune phase). Most of the complications occur in the immune phase and thus in the second week of the illness [1].

This illness is often forgotten and a high index of suspicion is required based not only on epidemiologic exposure but also on clinical findings consistent with the disease, as the reported case shows. First, a complete history and physical examination are key to an accurate diagnosis. The combination of nonspecific laboratory findings such as leukocytosis, thrombocytopenia, rhabdomyolysis, renal failure, and hepatic cytolysis and cholestasis with hyperbilirubinemia as presented in this case, can only suggest leptospirosis but not diagnose it [4]. Urinalysis frequently shows proteinuria, pyuria, granular casts, and occasionally microscopic haematuria. Although IgM ELISA is normally used as the standard serologic procedure, a negative result in a highly suspicious patient should not rule out the diagnosis since antibodies only become positive after the immune phase, between day five and seven of illness [5], being absent in the acute phase of the disease. Furthermore, antibodies can remain positive for several months, even years. PCR test can confirm the diagnosis in the early disease stage before the antibody titers are at a detectable level. PCR test is a rapid, sensitive, and specific test with the ability to assure a definite diagnosis during the acute phase of the disease [6].

Initial stabilization of patients with severe clinical leptospirosis involves managing the dehydration and shock with aggressive intravenous fluid therapy. In the context of a moderate-to-high clinical suspicion and the absence of a definitive laboratory diagnosis, initiation of empiric treatment is appropriate and a serologic positive test should not delay empiric treatment [1]. Doxycycline is effective both in eliminating leptospiremia and treating the carrier state.

The prognosis depends on the affected organs, the causative serovar, and evidently on individual patient characteristics and comorbidities. Mortality can vary from less than 5% to more than 15% among patients with severe illness [3], increasing when treatment is not initiated within 2-3 days of disease onset. In patients with severe pulmonary hemorrhagic syndrome, the mortality can exceed 50% [3]. An early diagnosis and appropriate treatment are crucial to improving the outcome, as this case proves.

## Conclusions

Leptospirosis is re-emerging as a new threat in developed countries so clinicians should be aware of this disease even outside endemic zones or without a major plausible exposure history. It remains a challenging diagnosis (and thus unreported) since it presents with marked clinical polymorphisms and clinicians must have a high degree of suspicion. Serological tests are often used to diagnose this disease albeit their low sensitivity. If there is a high clinical suspicion, a negative serological test should not be considered alone to rule out leptospirosis and prompt empirical antibiotic treatment to be initiated.
